# Cytokine Production by Human Colonic Intraepithelial Lymphocytes in Controls and Ulcerative Colitis

**DOI:** 10.1155/S0962935194000311

**Published:** 1994

**Authors:** P. Hoang, H. R. Dalton, H. J. de Silv, D. P. Jewell

**Affiliations:** 1 Service de Gastro-Enterologie Université Catholique de Louvain Bruxelles Belgium; 2 The Gastroenterology Unit Radcliffe Infirmary Oxford UK

## Abstract

Using an ELISA technique, concentrations of γ-interferon and interleukin-2 were assayed in the supernatants of colonic intraepithelial lymphocytes cultured with or without phytohaemagglutinin (PHA). IntraepitheHal lymphocytes produced low concentrations of γ-interferon and interleukin-2 when stimulated with PHA, but significantly more than when unstimulated (p < 0.05). There was no difference in production of these cytokines by IEL from control or inflammatory bowel disease.


Mediators of Inflammation 3, 219–222 (1994)

### Cytokine production by human colonic intraepithelial lymphocytes in controls and ulcerative colitis

P. Hoang,[1] H. R. Dalton,[2] H. J. de Silv[2] and D. P. Jewell[2,CA]

[1]Service de Gastro-Enterologie, Université Catholique de Louvain, Bruxelles, Belgium; [2]The Gastroenterology Unit, Radcliffe Infirmary, Oxford, UK

[CA] Corresponding Author

**Using an ELISA technique, concentrations of γ-interferon and interleukin-2 were assayed in the supernatants of colonic intraepithelial lymphocytes cultured with or without phytohaemagglutinin (PHA). Intraepithelial lymphocytes produced low concentrations of γ-interferon and interleukin-2 when stimulated with PHA, but significantly more than when unstimulated ($p < 0.05$). There was no difference in production of these cytokines by IEL from control or inflammatory bowel disease.**

**Key words:** γ-Interferon, Interleukin-2, Intraepithelial lymphocytes, Ulcerative colitis

### Introduction

Intraepithelial lymphocytes (IEL) are found interspersed between the epithelial cells of the gastrointestinal tract and so bear a close spatial relationship to luminal antigens. The precise function of IEL in the human is not known, although they may have a role in the induction of gut tolerance to luminal contents.[1,2]

Cytokines are soluble factors released by immune cells, especially macrophages and lymphocytes. One hypothesis for the pathogenesis of ulcerative colitis (UC) is that abnormal release of cytokines may result in disordered immunoregulation. Murine IEL do not spontaneously release interleukin-2 (IL-2) but do so when stimulated.[3–5] A γ-interferon-like factor has also been shown to be produced after stimulation with mitogens.[6]

Human small bowel IEL have been reported to produce IL-2 in response to stimulation with phytohaemagglutinin (PHA)[7] and γ-interferon (γIFN) in response to PHA and sheep red blood cell (RBC) lysates.[8] However, no data are available concerning cytokine production by human colonic IEL.

The aim of this study was to test the hypothesis that abnormal amounts of IL-2 and γIFN are produced by human colonic IEL in patients with UC.

### Materials and Methods

*Subjects*: Normal mucosa, at least 5 cm from any macroscopic lesion, from colonic resection specimens was used from nine control patients (eight carcinoma, one polyp; median age 68 years, range 38–81 years). The least inflamed segment of the resected colon was used from six patients with UC (median age 35 years, range 26–58 years). All the patients with UC underwent colonic resection for severe disease which was unresponsive to medical treatment and so were taking corticosteroids at the time of the study.

*Isolation of mucosal lymphocytes:* IEL were isolated as described previously.[1,2,7,8] Briefly, following resection, the colonic specimens were washed with Hanks' Balanced Salt Solution (Flow Laboratories, UK) without calcium and magnesium, and supplemented with gentamycin 50 μg/ml, penicillin 100 U/ml and streptomycin 100 μg/ml (HBSS). The mucosa was dissected and following further washing the mucosal strips were incubated with RPMI supplemented with 10% foetal calf serum, antibiotics and 1 mM dithiothreitol (Sigma) for 10 min. The mucosal strips were then washed a further three times in HBSS and incubated with 0.75 mM EDTA in a shaking water bath at 37°C for three periods of 30 min each at 140 oscillations per min. Following each incubation the supernatant containing the IEL was pooled, centrifuged at $500 \times g$ for 8 min and the pellet washed three times in RPMI. The cells were resuspended in RPMI and stored overnight at 4°C.

Further purification was achieved by passing this crude preparation down a glass wool column (Sigma) and performing a two-step Percoll gradient of 44% and 67.5%. The IEL were recovered from the interface.

*Monoclonal antibodies:* To detect T cell subsets, T helper/suppressor test (CD4 FITC, CD8 PE), LeucoGATE (HLe-1 FITC, Leu-M3 PE) and simultest

© 1994 Rapid Communications of Oxford Ltd control reagent ($IgG_1$, FITC, $IgG_2$ PE) were used. All monoclonal antibodies were prepared by Becton Dickinson Immunocytometry Systems (Mountain View, CA, USA).

*Immunofluorescence and FACS analysis:* Immunofluorescent staining was performed as described previously. Briefly, cells were suspended in PBS/0.1% sodium azide to a concentration of $10^6$ cells/ml. Aliquots of 100 μl were incubated with 8 μl of monoclonal antibody in the dark at 4°C for 45 min. The cells were then washed with PBS, centrifuged and resuspended in 500 μl PBS, paraformaldehyde 1%. Flow cytometric analysis was performed using a FACScan (Becton Dickinson).

*Preparation of lymphokine-containing supernatant:* IEL at a concentration of $1 \times 10^6$ were cultured in 1 ml aliquots in 24-well plates (Linbro, Flow Laboratories, UK) with or without 10 μg/ml PHA (Wellcome Laboratories, Beckenham, UK) for 72 h at 37°C and 5% $CO_2$. The supernatants were then harvested, centrifuged and passed through a 0.22 μm filter and stored at −20°C until analysis was performed.

*Cytokine assays:* γIFN was measured by an enzyme amplified sensitivity immunoassay of proven specificity (Medgenix Diagnostics, Belgium). IL-2 was measured by a sandwich enzyme immunoassay of proven specificity (T Cell Sciences, Cambridge, MA, USA). Optical densities were measured by an automated dual beam ELISA reader (Titertek Multiscan, Flow Laboratories, UK) at 450 nm for γIFN and 490 nm for IL-2. Concentrations of the cytokines were determined by reference to the serially diluted standards included on each plate. The minimum detectable concentrations of these assays were: γIFN, 0.03 U/ml; IL-2, 6 pg/0.1 ml. Recombinant human γIFN and IL-2 were used as positive controls.

*Culture of HT29 for Class II induction:* γIFN induces Class II expression on the colonic cancer cell line HT29 in a dose-dependent fashion.[9] Serial dilutions of supernatants from IEL cultured with or without 10 μg/ml PHA were cultured with $1 \times 10^6$ HT29 cells (a generous gift of Dr. P. Brandtzaeg) for 72 h in a 24-well plate at 37°C in 5% $CO_2$. The HT29 cells were then stained with 10 μl of phycoerythrin anti-HLA DR monoclonal antibody (Becton Dickinson, Mountain View, CA, USA) and analysed by flow cytometry (FACScan, Becton Dickinson).

*Ethics:* Ethics Committee approval for this study was obtained from the Central Oxford Regional Ethics Committee.

*Statistics:* The Mann–Whitney *U* and Spearman's Rank Correlation tests were used when appropriate. $p < 0.05$ was taken as significant.

### Results

*Phenotypic characterization of mucosal lymphocytes:* The IEL populations had a median CD4/CD8 ratio of 0.22 (controls, range 0.10–0.33) and 0.20 (UC, range 0.12–0.30).

*Lymphokine production by PHA-stimulated colonic IEL:* The median concentration of γIFN secreted by IEL alone (control *vs.* UC) was 0.3 U/ml (range 0–0.6) and 0.5 U/ml (range 0–0.7) respectively (not significant). Following stimulation with PHA the concentration in controls increased to 3.9 (range 0.9–26) and in UC to 0.85 (range 0.3–10.1). These values were significantly higher than for the unstimulated IEL ($p < 0.05$, Fig. 1). The median concentration of IL-2 secreted by IEL alone (control *vs.* UC) was 1 U/ml (range 0–20) and 0 U/ml (range 0–10) respectively (not significant). Following stimulation with PHA the concentration in controls increased to 15 U/ml (range 0–30) and in UC to 5.5 U/ml (range 0–50).

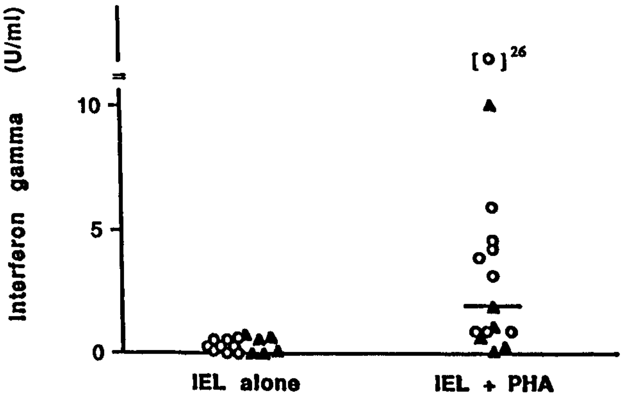

FIG. 1. γ-interferon (γIFN) concentrations (U/ml) secreted by unstimulated IEL and IEL cultured with 10 μg/ml PHA from controls and ulcerative colitis. γIFN production is significantly higher from IEL cultured with PHA ($p < 0.05$). UC *vs.* controls, not significant. ▲, UC; O, controls.

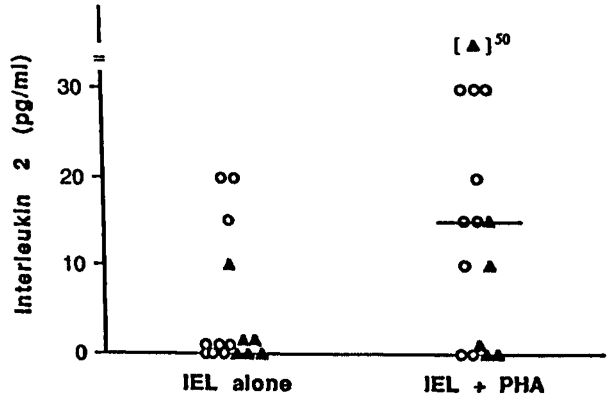

FIG. 2. Interleukin 2 (IL-2) concentration (pg/ml) secreted by IEL from controls and ulcerative colitis cultured with and without 10 μg/ml PHA. IL-2 production is significantly higher from IEL cultured with PHA ($p < 0.05$). UC *vs.* controls, not significant. ▲, UC; O, controls.

These values were significantly higher than for the unstimulated IEL ($p < 0.05$, Fig. 2).

*Induction of HLA–DR molecules by culture supernatant on HT29:* Supernatants of unstimulated IEL when incubated with HT29 cells produced no detectable expression of HLA–DR molecules, as assessed by flow cytometry. However, the supernatants of stimulated control IEL produced 5% HLA–DR expression of HT29 (Fig. 3). This effect was also observed using supernatants of stimulated IEL which had been irradiated (2 500 Rad) prior to culture (data not shown).

### Discussion

Rat IEL produce a γIFN-like factor capable of producing Ia expression on IEC 17 cells.[6] Lymphocyte proliferation was not essential for the production of this factor. In mice, Con A stimulated IEL to produce moderate levels of γIFN.[4] Human small intestinal IEL have been reported to produce γIFN after stimulation with PHA.[8] This production of γIFN is increased many times by the addition of sheep RBC lysates.

The present data show that colonic IEL produce very low amounts of γIFN. Although there was a significant increase in γIFN production following stimulation with PHA, the absolute amounts of γIFN produced were persistently low. This is not due to the failure of lectin binding.[10] The amount of γIFN produced by stimulated IEL is of sufficient quantity to induce Class II molecules on HT29 cells. Class II bearing epithelial cells can in turn cause further activation of IEL.[11] In contrast to a previous study on cytokine production by lamina propria lymphocytes there was no significant difference in γIFN production by IEL between control and IBD patients.[12]

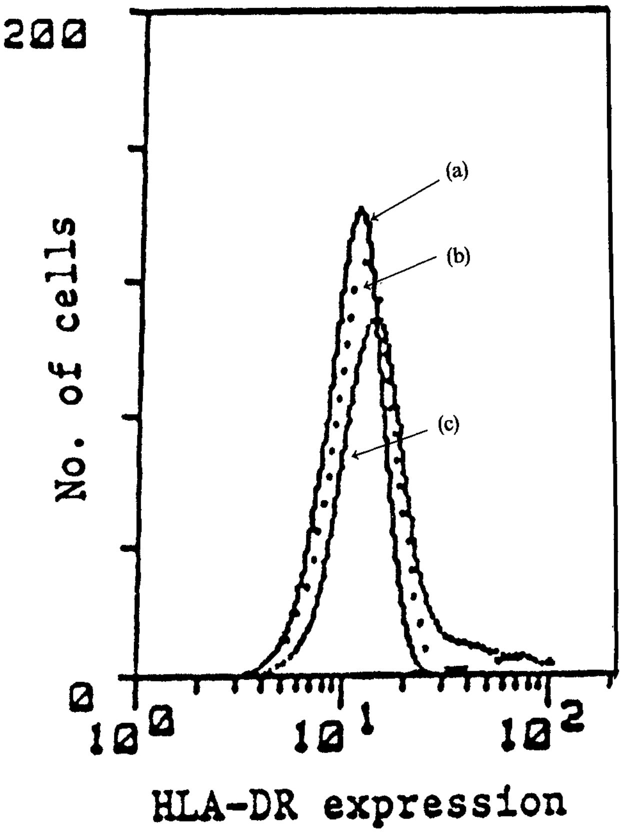

FIG. 3. Flow cytometric analysis of the effect of mucosal lymphocyte supernatants on HLA–DR expression by HT29 cells. —— (a), HT29 alone (control); ····· (b), HT29 + supernatant of unstimulated IEL; ······· (c), HT29 + supernatant of stimulated IEL.

Most animal studies have shown that IEL do not produce IL-2. Human small bowel IEL produce similar amount of IL-2 to peripheral blood T cells following stimulation with mitogen.[7] These data confirm the finding that human colonic IEL produce IL-2. In addition we have shown that there is no difference in IL-2 production by colonic IEL in control and UC patients.

It has recently been proposed that helper $T_H$ ($CD4^+$) cells can be classified with relationship to their cytokine production profile.[13] Thus, $T_H$ cells which produce small amounts of IL-2 and γIFN but large amounts of IL-4, IL-5 and IL-10 are classified as $T_H2$ cells. Conversely, cells producing large quantities of IL-2 and γIFN but little IL-4, IL-5 and IL-10 are called $T_H1$ cells.

Human $CD8^+$ clones have also been analysed in a similar manner. Lepromin specific $CD8^+$ T suppressor clones from patients with lepromatous leprosy produce IL-4 and little or no γIFN (Type 2 pattern of lymphokine production). In contrast, alloreactive HLA B27-specific human $CD8^+$ CTL clones produce γIFN but no IL-4 (Type 1 lymphokine pattern). In these experiments, suppression was IL-4 dependent and not necessarily related to phenotype, as $CD4^+$ clones which secreted IL-4 also caused suppression.[14,15]

Human colonic IEL have recently been shown to have potent suppressive properties on the proliferative responses of autologous LPL.[1,2] This effect was found to be CD8-dependent, γδ-independent and mediated by a soluble factor. The data presented in this paper indicate that IEL produce only small quantities of γIFN and IL-2, which indicates that they could have a Type 2 pattern of lymphokine production. Were this to prove to be the case, it may explain the potent down-regulatory properties of IEL, on the basis of IL-4 and/or IL-10 secretion.

In conclusion, colonic IEL produce the cytokines γIFN and IL-2, although in small amounts. There is no significant difference in production of γIFN and IL-2 in control and UC patients.

### References

1. Hoang P, Crotty B, Dalton HR, Jewell DP. Human colonic intraepithelial lymphocytes are suppressor cells. *Clin Exp Immunol* 1991; **85**: 498–503.
2. Dalton HR, Di Paolo MC, Sachdev GK, Crotty B, Hoang P, Jewell DP. Human colonic intraepithelial lymphocytes from patients with inflammatory bowel disease fail to down-regulate proliferative responses of primed allogeneic peripheral blood mononuclear cells after rechallenge with antigens. *Clin Exp Immunol* 1003; **93**: 97–102.

3. Dillon SB, MacDonald TT. Functional characterisation of con-A responsive Lyt 2-positive mouse small intestinal intraepithelial lymphocytes. *Immunology* 1986; **59**: 389–396.
4. Dillon SB, Dalton BJ, MacDonald TT. Lymphokine production by mitogen and antigen activated mouse intraepithelial lymphocytes. *Cell Immunol* 1986; **103**: 326–338.
5. Mowat MA, McInnes IB, Parrott DMV. Functional properties of intraepithelial lymphocytes from mouse small intestine IV. Investigation of the proliferative capacity of IEL using phorbol ester and ionophore. *Immunology* 1989; **66**: 398–403.
6. Cerf-Bensussan N, Quaroni A, Kurnick JT, Bhan AK. Intraepithelial lymphocytes modulate 1a expression by intestinal epithelial cells. *J Immunol* 1986; **132**: 2244–2251.
7. Ebert EC, Roberts AL, Brolin RE, Raska K. Examination of the lower proliferative capacity of human jejunal intraepithelial lymphocytes. *Clin Exp Immunol* 1986; **65**: 148–157.
8. Ebert EC. Intraepithelial lymphocytes: interferon-γ production and suppressor/cytotoxic activities. *Clin Exp Immunol* 1990; **82**: 81–85.
9. Kvale D, Brandtzaeg P, Lovhaud D. Up-regulation of the expression of secretory component and HLA molecules in a human colonic cell line by tumour necrosis factor-α and gamma interferon. *Scand J Immunol* 1988; **28**: 351–357.
10. Greenwood JH, Austin LL, Dobbins WO. *In vitro* characterisation of human intraepithelial lymphocytes. *Gastroenterology* 1983; **85**: 1023–1035.
11. Hoang P, Crotty B, Dalton HR, Jewell DP. Epithelial cells bearing Class II molecules stimulate allogeneic human colonic intraepithelial lymphocytes. *Gut* 1992 (in press).
12. Lieberman BY, Fiocchi C, Youngman KR, Spatnekar WK, Proffitt MR. Interferon-γ production by human intestinal mucosal mononuclear cells. Decreased levels in inflammatory bowel disease. *Dig Dis Sci* 1988; **33**: 1297–1304.
13. Mosmann TR, Moore KW. The role of IL-10 in crossregulation of $T_H1$ and $T_H2$ responses. *Immunology Today* 1991; **12**: A49–A52.
14. Salgame PR, Abrams JS, Clayberger C *et al.* Differing lymphokine profiles of functional subsets of human CD4 and CD8 T cell clones. *Science* 1991; **254**: 279–282.
15. Bloom SR, Salgame P, Diamond P. Revising and revisiting suppressor T cells. *Immunol Today* 1992; **13**: 131–136.

ACKNOWLEDGEMENT. Dr P. Hoang was in receipt of a grant from the National Association for Colitis and Crohn's Disease.

**Received 18 January 1994; accepted in revised form 25 March 1994**
